# Impact of emotional intelligence on academic procrastination among EFL learners in China: Serial mediation role of self-efficacy and psychological resilience

**DOI:** 10.3389/fpsyg.2026.1693897

**Published:** 2026-03-10

**Authors:** Li Li, Xianli Gao

**Affiliations:** 1 School of Foreign Studies, Guangzhou University, Guangzhou, China; 2 School of Foreign Languages, Guangdong Pharmaceutical University, Guangzhou, China

**Keywords:** academic procrastination, China, EFL learners, emotional intelligence, psychological resilience, self-efficacy

## Abstract

Procrastination is a phenomenon that has a double connotes, positive and negative and can occur in all individuals. There are many factors that cause individuals to procrastinate their works, both situational factors and personal factors. Few studies have focused on the impact of individuals’ emotional intelligence on their academic procrastination, especially among university students in the context of Chinese higher education. Drawing upon this existing research gap, the present study set forth to test a serial mediation model of Chinese universities students’self-efficacy and psychological resilience in China. The Emotional Intelligence Scale, general Self-efficacy Scale, brief Psychological Resilience Scale, and Academic Procrastination Scale were administered to 880 EFL learners in Chinese universities. The results of this study indicated that: (a) Academic procrastination is negatively correlated with emotional intelligence, self-efficacy, and psychological resilience; (b) Emotional intelligence is a significant negative predictor of academic procrastination; (c) Self-efficacy and psychological resilience partially mediate the link between emotional intelligence and academic procrastination, and jointly create a serial mediating effect. The results reveal the pathway through which internal individual factors affect academic procrastination, and highlight the significance of learners’ psychological resources in affecting academic procrastination, which offers fresh perspectives and approaches for understanding and addressing college students’ academic procrastination. Detailed discussion is presented in this study.

## 1 Introduction

Procrastination unnecessarily puts off everyday jobs to the point that one starts to feel uneasy (Solomon and Rothblum, 1984), and can be permanent or temporary and defined as a rationale for delaying behavioral or cognitive output, making a decision, or taking an action (Song et al., 2022). Academic procrastination is the concrete manifestation of procrastination within the academic domain. At its core, it is the voluntary avoidance of learning tasks and the failure of self-regulation in the learning process (Senecal et al., 1995), which is characterized by irrational delay, and even when ample time is available, the individual keeps postponing the intended tasks until the deadline forces action (Steel, 2007). Academic procrastination is prevalent among university students. Those who procrastinate seem to delay and postpone their academic work, becoming self-explanatory and disregarding their academic obligations during their whole study period (Gutiérrez-García et al., 2020). Academic procrastination denotes a delay in academic activity in education and training (Vargas, 2017), and may be deliberate, unintentional, or habitual, but it substantially impacts university students’ learning and success (Hussain and Sultan, 2010). Asri et al. (2017) found that students with high academic procrastination have weak personality traits, negative self-image, lack of self-regulation, high anxiety attitudes, weak cognitive, negative learning experiences, weak mental and physical conditions. Consequently, reducing academic procrastination is crucial for improving the effectiveness of higher education.

Ultimately, academic procrastination not lies in learning burnout or time-management, but in a complex psychological process involving cognition, emotion, and behavior (Solomon and Rothblum, 1984). Emotion, as a moderator between cognition and behavior, plays a critical role. Specifically, an individual’s capacity for emotional regulation-that is, emotional intelligence, significantly influences procrastination (Wang, 2019). Emotional intelligence, also termed emotional–social intelligence, denotes a positive psychological disposition that integrates emotional and social competencies. It enables effective expression and appraisal of one’s own and others’ emotions, regulation of emotions across contexts, and the constructive use of emotion to solve problems (Zhang and Zhang, 2025). In addition, emotional intelligence is a robust predictor of academic procrastination. Previous research has uncovered a potential mechanism-that is, procrastination can be viewed as a failure of emotional regulation. Learners delay tasks to temporarily reduce negative effect, yet this only generates more negative emotion, thereby intensifying procrastination. Effectively emotional regulation, conversely, serves as a protective factor against it (Luo, 2023). Although current evidence demonstrates a close link between emotional intelligence and academic procrastination, the internal mechanisms remain unclear. Hence, the present study introduces self-efficacy and psychological resilience to explore the issue further from the perspective of positive psychology.

Self-efficacy, defined by Bandura (1997) as the personal capacity to cope with specific situations, is a determining psychological variable strongly predictive of academic achievement (Feldman and Kubota, 2015; Pajares and Schunk, 2001). This is because self-efficacy involves beliefs about one’s capacity to organize and execute actions to achieve specific results (Contreras, 2005). Regulatory Emotional Self-Efficacy Theory posits that confidence in managing emotions directly affects behavioral persistence. Individuals with higher emotional intelligence, by improving their emotional regulation skills, strengthen the belief that “I can do it,” thereby lowering the resistance to task initiation (Bandura and Locke, 2003). Some previous empirical studies support this pathway-that is, self-efficacy mediates 37% of the effect of emotional intelligence on academic procrastination, and this effect is consistent across disciplines (Linda T. et al., 2004). Structural equation modeling shows that the indirect effect via self-efficacy dominates over the direct effect of emotional intelligence on procrastination (Liu, 2014). Temporal Motivation Theory further suggests that under high stress, the buffering role of self-efficacy is amplified, and the inhibitory effect of emotional intelligence on procrastination relies more heavily on this mediating path. Moreover, recent work has expanded the complexity of the mediation. Nathan et al. (2019), using an inter-actionist model, found that emotional intelligence inhibits procrastination indirectly through self-efficacy, while its direct effect remains significant. Liu (2014) revealed that emotional regulation difficulties influence procrastination via academic self-concept, with self-efficacy acting as a moderator in this pathway. Limone et al. (2020) integrated multiple paths into a comprehensive model in which self-efficacy accounts for 42% of the mediation, operating through serial effects involving learning motivation and time management. Furthermore, self-efficacy indirectly reduces procrastination by influencing learners’ motivation, time-management skills, and emotional states (Sarvenaz et al., 2021). Accordingly, based on the above arguments and prior empirical support, the following hypothesis is advanced:

H1: Self-efficacy mediates the relationship between emotional intelligence and academic procrastination of EFL Learners in Chinese universities.

One specific theory that may help to further explain the relationship between emotional intelligence and learner academic outcomes is Fredrickson’s (2001) Broaden and Build Theory. Through this theory, it is posited that positive emotions broaden one’s mindset resulting in increased attention to learning. Conversely, negative emotions are thought to narrow cognitions and hinder learning (Fredrickson, 2001). Building on this theoretical perspective, researchers have begun to advocate the development of the personal characteristic of psychological resilience as it has been identified as a partial mediator of enhanced growth mindsets. Psychological resilience refers to the dynamic capacity by which individuals maintain psychological equilibrium, adapt to their environment, and achieve growth when confronted with prolonged stress or significant setbacks. Specifically, psychological resilience is thought to support an individual’s efforts to effectively cope with stressors that have the potential to promote motivation and positive action (Fletcher and Sarkar, 2012; Frank and R. Kodikal, 2019). Previous studies indicated that resilience is significantly and negatively correlated with academic procrastination (Luthar and Cicchetti, 2000), that is, students with higher academic resilience cope better with stress, sustain positive learning attitudes, and consequently procrastinate less. Emotional intelligence is positively related to psychological resilience, and the two exert a synergistic effect in reducing academic procrastination (Cai, 2024). On the one hand, emotional intelligence helps learners regulate their emotions, thereby enhancing resilience; on the other hand, resilience enables learners to maintain a positive mindset under academic stress, which in turn further elevates emotional intelligence. This mutually reinforcing relationship assists learners in meeting academic challenges and curbing procrastination. Thus, based on the above arguments, this study offers the following hypothesis:

H2: Psychological resilience mediates the relationship between emotional intelligence and academic procrastination of EFL Learners in Chinese universities.

Psychological resilience is also closely linked to self-efficacy. Social Cognitive Theory posits that high self-efficacy strengthens persistence under pressure (Bandura, 1986). And this trait is the core driver of psychological resilience. Yu and Zhang (2013) viewed that self-efficacy reinforces resilience through a “goal-action-feedback” loop-that is, as students continually invest effort in academic challenges, accumulated successes boost self-efficacy, which in turn fortifies confidence in coping with setbacks. Previous studies revealed that students with higher resilience are more inclined to set challenging learning goals, and the process of goal attainment continually elevates their self-efficacy (Hui and Feng, 2023), while high self-efficacy indirectly reduces procrastination by enhancing emotional regulation skills (Zhou, 2022). Individuals with strong self-efficacy tend to use cognitive reappraisal, reframing academic setbacks as opportunities for growth, which directly strengthens their resilience (Linda T. et al., 2004). By breaking goals into sub-goals and accumulating small successes, self-efficacy creates a virtuous cycle of “micro-achievement → efficacy enhancement → resilience strengthening” (Luo, 2023). Thus, this study offers the following hypothesis based on the above arguments and available empirical support:

H3: Psychological resilience and self-efficacy act as a serial mediator between emotional intelligence and academic procrastination of EFL Learners in Chinese universities.

## 2 Method

### 2.1 Participants

Convenience sampling was recruited via the online platform *Wenjuanxing*.^1^ Questionnaires were distributed to EFL Learners at 30 universities in South China. Over 1,000 potential participants were contacted via email, informing them that they would need to complete an online questionnaire at three time points in 2023–2024 academic year, and inviting them to volunteer their participation. A total of 951 responses were returned. After excluding invalid cases (straight-lining responses, identical answers, duplicate IP addresses), 880 valid questionnaires were finally retained (effective response rate = 92.5%). The final sample comprised 303 male (34.4%), and 577 female (65.6%), and first-year 199 (22.6%), second-year 189 (21.5%), third-year 242 (27.5%), and fourth-year 250 (28.4%). And mean age is 19.6 years (SD = 0.91). At the time of questionnaire administration, all participating learners were in full-time education.

### 2.2 Instruments

Four questionnaires, including a socio-demographic data sheet, were used in the present study to collect self-report data. The tools used were psychometrically reliable and valid. Original questionnaires were in English, and a Chinese-translated version of the tools was used for data collection.

#### 2.2.1 Emotional intelligence scale

Emotional Intelligence Scale, revised by Wang et al. (2021) from the original developed by Wong and Law’s (2004), was employed in the present study. It is a 16-item scale that measures one’s emotional intelligence in four different dimensions (4 items each): (a) appraisal and expression of one’s own emotions, (b) recognition of others’ emotions, (c) regulation of one’s own emotions, and (d) use of emotion to self-motivate. All items were measured with indicators on a Likert-type 7-point scale (1 = completely disagree to 7 = completely agree). Higher total scores indicate greater emotional intelligence with Cronbach’s α 0.92 overall and sub-dimension Cronbach’s α ranging from 0.83 to 0.89.

#### 2.2.2 General self-efficacy scale

General Self-Efficacy Scale, revised by Wang et al. (2001) from the original developed by Schwarzer & Schwarzer and Born (1997), was used in the present study. It is the single-dimension scale with 10 items that measure self-efficacy on 4-point Likert-type response patterns ranging from 1 (not at all true) to 4 (exactly true). Low scores indicate low levels, and high scores indicate high levels of self-efficacy. Scores can range from 10 to 40. The study has reported the reliability of self-efficacy with Cronbach’s α 0.89.

#### 2.2.3 Brief resilience scale

Brief Resilience Scale, revised by Yu and Zhang (2013) from the original developed by Connor (2006), was used in the present study. It is a 25-item scale covering three dimensions: tenacity, strength, and optimism to measure the psychological resilience of the participants. Higher total scores indicate greater psychological resilience with Cronbach’s α 0.91 overall and sub-dimension Cronbach’s α ranging from 0.84 to 0.87.

#### 2.2.4 Academic procrastination scale

College Student Academic Procrastination Scale, revised by Pang and Han (2009) from the original developed by McCloskey and Scielzo (2015), was used in the present study. It is a 37-item scale that measures college students’ emotional intelligence in three different dimensions: assignment completion, exam preparation and self-directed learning. The scale preamble asked students to indicate how frequently last week they engaged in the following behaviors or thoughts on a 5-point Likert scale ranging from 1 (never) to 5 (always). Higher scores indicate greater procrastination with Cronbach’s α 0.93 overall and sub-dimension Cronbach’s α ranging from 0.89 to 0.92.

### 2.3 Statistical analysis

The primary data analysis used frequency, percentages, mean, and standard deviation. The product–moment coefficients were calculated to determine the magnitude and direction of the relationship between study variables. In addition, various other preparatory analyses, including reliability, were determined using SPSS version 26. A confirmatory factor analysis of the variables used in this study was conducted to develop a measurement model with an acceptable model fit. Pearson correlations examined initial associations among variables. Structural equation modeling was conducted by the maximum-likelihood method in the AMOS version 24 to test the mediating roles of self-efficacy and psychological resilience. Emotional intelligence, self-efficacy and psychological resilience were modeled as independent variables, and academic procrastination was dependent one. The model fit indices, also known as measures of goodness of fit, help us to scrutinize the observed model by comparing it with the theoretical model. Widely used fit measures are normed chi-square, Probability, root mean square error of approximation (RMSEA), comparative fit index (CFI), goodness of fit index (GFI), adjusted goodness of fit index (AGFI), and parsimony goodness of fit index (PGFI). In this study, model fit was evaluated with CFI, TLI, RMSEA, and GFI (acceptable: RMSEA<0.08, CFI ≥ 0.90, TLI ≥ 0.90, GFI ≥ 0.90). Moreover, indirect effects were examined using a bootstrap approach with 5,000 resamples, generating bias-corrected 95% confidence intervals. Effects were deemed significant if the 95% CI did not include zero (Preacher and Hayes, 2008). Mediation analyses were carried out for further research.

## 3 Results

### 3.1 Common method bias

Generally, survey-based research has inherent common method bias (CMB). Although anonymity and temporal separation were used to minimize common source bias in this study, residual bias could still exist because all data came from a single source. Therefore, it is necessary to statistically check the extent of the CMB. Three tests were conducted: (a) We followed Harman’s single-factor method and found that a single factor accounted for 21.63 percent of the variance; (b) we verified the variance inflation factor (VIF) and found that the VIF values were less than 5.0; and (c) we conducted latent variable approach by subjecting all the constructs on a single factor and rotated with all constructs and found that the inner VIF values were less than 3.3 (Kock, 2015). These statistics reveal that CMB did not affect the data.

### 3.2 Descriptive statistics and correlation analysis

Before testing the relationship model, testing the relationship between variables used in the research needs to be done by using correlation analysis. Correlations were also used to ensure that there was no multicollinearity between the independent variables used in this study. The correlation between variables or constructs used in this study, means, and standard deviations are presented in Table 1. Based on Table 1, certain correlations exist among the variables used in this study. Specifically, academic procrastination is negatively correlated with emotional intelligence (r = −0.691, *p* < 0.01), self-efficacy (r = −0.732, p < 0.01), and psychological resilience (r = −0.597, p < 0.01), the strongest link being with self-efficacy. In addition, emotional intelligence was moderately positively related to self-efficacy (r = 0.583, p < 0.01) and strongly positively related to resilience (r = 0.702, *p* < 0.001); self-efficacy and resilience were also strongly positively correlated (r = 0.814, p < 0.01) (see Table 1).

**Table 1:** Mean, standard deviation, and inter correlations among all variables.

Variables	*M*	*SD*	1	2	3	4
1. Emotional intelligence	34.43	6.30	1			
2. Self-efficacy	41.83	6.43	0.583^**^	1		
3. Psychological resilience	47.72	12.67	0.702^**^	0.814^***^	1	
4. Academic procrastination	55.49	15.37	−0.691^**^	−0.732^***^	−0.597^**^	1

** Correlation is significant at the 0.01 level (2-tailed).

*** Correlation is significant at the 0.001 level (2-tailed).

Sources: Primary Data, processed.

### 3.3 Mediation analysis

To address the mechanism, serial mediation was tested using a mediation analysis via Model 6 of PROCESS macro (Hayes and Heit, 2018). The model specification included emotional intelligence as the predictor, academic procrastination as the outcome, and self-efficacy (Mediator 1) and psychological resilience (Mediator 2) as sequential mediators. Model testing results indicated that emotional intelligence significantly predicted self-efficacy (β = 0.612, t = 18.782, p < 0.001) and psychological resilience (β = 0.302, t = 5.471, p < 0.001). Self-efficacy significantly predicted psychological resilience (β = 0.369, t = 6.852, p < 0.001) and negatively predicted academic procrastination (β = −0.294, t = −4.081, p < 0.01). Psychological resilience negatively predicted academic procrastination (β = −0.408, t = −7.751, p < 0.001). The direct effect of emotional intelligence on academic procrastination became non-significant after the mediators were entered (β = −0.107, t = 0.438, *p* > 0.05) (see Table 2).

**Table 2:** Regression analysis of relationships of all variables.

Regression equation		Goodness-of-fit measures			Significance testing	
Outcome	Predictor	*R*	*R^2^*	*F*	β	*t*
SE	EI	0.455	0.210	306.950	0.612	18.782^***^
PR	EI	0.404	0.154	117.040	0.302	5.471^***^
	SE				0.369	6.852^***^
AP	EI	0.308	0.089	89.028	−0.107	0.438
	SE				−0.294	4.081^**^
	PR				−0.408	−7.751^***^
AP	EI	0.212	0.054	30.067	−0.223	−5.491^***^

** Correlation is significant at the 0.01 level (2-tailed).

*** Correlation is significant at the 0.001 level (2-tailed).

SE, Self-efficacy; PR, psychological resilience; AP, academic procrastination; EI, emotional intelligence. Sources: Primary Data, processed.

**Figure 1:**
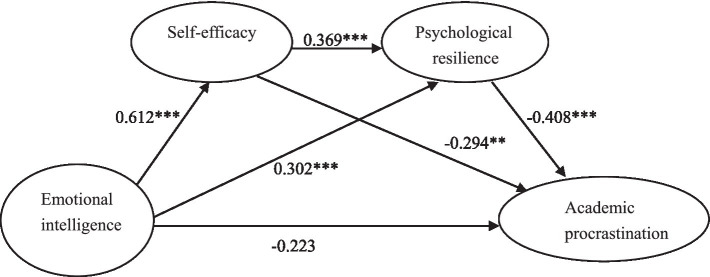
Serial mediation model.

Through further mediation analysis, it was found that a partial mediation exists between self-efficacy and academic procrastination. The mediation effect size of self-efficacy was −0.402, indicating it accounted for 118.24% of the total effect (−0.340) between emotional intelligence and academic procrastination. Additionally, Bootstrapping analysis with 5,000 resamples was used to examine the mediation effects and confidence intervals. As shown in Table 3, the mediation effect between emotional intelligence and academic procrastination consists of three indirect pathways: (a) Path 1″Emotional Intelligence → Self-efficacy → Academic Procrastination” had a mediation effect of −0.135, accounting for 39.71% of total effect with 95% CI [−0.238, −0.045]. The confidence interval excluded zero, indicating a significant mediation coefficient; (b) Path 2″Emotional Intelligence → Psychological Resilience → Academic Procrastination” showed a mediation effect of −0.159, accounting for 46.76% of total effect with 95% CI [−0.228, −0.131]. The confidence interval excluded zero, demonstrating a significant mediation coefficient; (c) Path 3″Emotional Intelligence → Self-efficacy → Psychological Resilience → Academic Procrastination” yielded a mediation effect of −0.108, accounting for 31.76% of total effect with 95% CI [−0.149, −0.058]. The confidence interval excluded zero, demonstrating a significant mediation coefficient (Preacher and Hayes, 2008). The direct effect of emotional intelligence on academic procrastination remained significant even after introducing the mediating variables of self-efficacy and psychological resilience. Thus, self-efficacy and psychological resilience serve as partial mediators between emotional intelligence and academic procrastination. This mediation model is presented in Figure 1.

**Table 3:** Test results of mediation model based on the bootstrap method.

Path of effects	Value	Boot *SE*	95% CI		Relative effect
			Boot LL	Boot UL	
Total effect	−0.340	0.071	−0.499	−0.239	
Direct effect	0.062	0.082	−0.108	0.200	−18.24%
Total indirect effect	−0.402	0.061	−0.516	−0.303	118.24%
Path 1: EI→SE→AP	−0.135	0.054	−0.238	−0.045	39.71%
Path 2: EI→PR→AP	−0.159	0.037	−0.228	−0.131	46.76%
Path3: EI→SE→PR→AP	−0.108	0.027	−0.149	−0.058	31.76%

EI, emotional intelligence; SE, self-efficacy; PR, psychological resilience; AP, academic procrastination. Sources: Primary Data, processed.

## 4 Discussions

The study extends the present literature by examining the effects of emotional intelligence on academic procrastination among EFL learners in Chinese universities. Further, it also investigated the serial mediation role of self-efficacy and psychological resilience. Empirical results indicate that emotional intelligence is significantly associated with self-efficacy, psychological resilience, and academic procrastination, consistent with previous findings. Further mediation effect tests reveal that emotional intelligence not only exerts a direct impact on EFL learners’ academic procrastination but plays an indirect impact through the mediating roles of psychological resilience and self-efficacy. To be specific, this mediation model includes three pathways: (a) the path using self-efficacy as a mediator, (b) the path using psychological resilience as a mediator, and (c) the path featuring a serial mediation effect from self-efficacy to psychological resilience.

### 4.1 Relationship between emotional intelligence and academic procrastination

The results show that emotional intelligence has negative effects on EFL learners’ academic procrastination-that is, higher emotional intelligence can predict fewer procrastination behaviors, which is in line with Magnano et al. (2016). Emotionally intelligent EFL learners with advanced self-emotion regulation and emotional utilization skills place particular emphasis on self-awareness and the perception of thoughts and emotions, which profoundly impacts academic procrastination. Those with higher emotional intelligence demonstrate greater confidence in their ability to perceive and regulate emotions. Consequently, they exhibit healthier physiological reactions and heightened resilience, thus being less likely to postpone learning tasks due to negative emotions such as anxiety or fear. As evidenced by Wang and Li (2021), university students generally exhibit relatively high emotional intelligence, reflecting positive influences from education systems, family environments, and societal climates, and providing strong support for social adaptation, mental health, and career development. However, this does not mean that individual differences and the dynamic nature of emotional intelligence can be overlooked. Continuously monitoring and cultivating EFL learners’ emotional intelligence remains a vital duty for both education and society. Recent years have witnessed a growing body of research on group-based interventions or training programs for enhancing emotional intelligence among university students. Previous studies indicate that emotional intelligence group training can elevate students’ positive emotions, reduce procrastination, bolster self-efficacy, enabling students to focus more effectively on academic tasks (Wen et al., 2014). Although emotional intelligence exerts a clear direct inhibitory effect on procrastination, its predictive power is significantly amplified when combined with self-efficacy and resilience. This phenomenon underscores that the efficacy of emotional intelligence in mitigating academic procrastination demands not only its intrinsic inhibitory function but more crucially synergistic effects mediated by self-efficacy and resilience mechanisms. Without systematic cultivation of these mediating factors, emotional intelligence alone would yield significantly constrained inhibitory outcomes.

### 4.2 Relationship between emotional intelligence and academic procrastination moderated separately by self-efficacy and by psychological resilience

The results indicate that self-efficacy shows a significant positive correlation with emotional intelligence, while exhibiting a negative correlation with academic procrastination. Furthermore, the mediating pathway of “emotional intelligence → self-efficacy → academic procrastination” is significant, which means that emotional intelligence not only directly affects academic procrastination but also indirectly influences it through self-efficacy, confirming Hypothesis 1. The significance of this mediating pathway lies in a bidirectional reinforcement system, where emotional intelligence inhibits academic procrastination by enhancing an individual’s self-efficacy.” On one hand, emotional intelligence positively predicts EFL learners’ self-efficacy, directly reinforcing it. Individuals with high emotional intelligence can effectively manage negative emotions such as anxiety and frustration, reducing emotional exhaustion. This stable emotional state enables individuals to focus more on tasks themselves rather than being trapped by fear of failure, thereby strengthening their confidence in their own abilities. The emotion-understanding component of emotional intelligence facilitates rational judgment of tasks. By transforming negative attributions like “I cannot do this” into positive cognitions such as “I need to adjust my strategies,” individuals develop efficacy beliefs of “I can control my learning progress” (Baumeister, 2007). Adaptive behaviors supported by emotional intelligence—such as proactively seeking help or creating study plans—accumulate concrete successful experiences. These micro-achievements continuously reinforce self-efficacy through the emotion-behavior-outcome cycle. On the other hand, EFL learners’ self-efficacy negatively predicts their academic procrastination and actively suppresses it. According to Resource Constraints Theory, diminished self-control and reduced self-efficacy trigger various procrastination behaviors, with academic procrastination being a prominent manifestation (Baumeister, 2007). When facing academic setbacks, high self-efficacy buffers against negative emotions by associating learning tasks with self-capability validation. This linkage generates intrinsic motivation, prompting individuals to choose immediate action over escape-delay patterns (Wang and Gao, 2021). Even when temporary procrastination occurs, individuals rapidly resume interrupted tasks driven by their conviction in self-competence, preventing chronic procrastination. This dynamic regulatory mechanism proves particularly potent in high-pressure learning contexts. Consequently, enhancing self-efficacy is paramount for mitigating academic procrastination.

Empirical analysis further reveals a moderate positive correlation between emotional intelligence and psychological resilience, while psychological resilience demonstrates a significant inhibitory effect on academic procrastination. More critically, mediation effect tests support the pathway model of “emotional intelligence → psychological resilience → academic procrastination.” This indicates that emotional intelligence not only directly reduces academic procrastination but also indirectly mitigates procrastination by enhancing psychological resilience, thereby confirming Hypothesis 2. The significance of this mediating pathway is reflected in the integrated effect of a dual-conduction mechanism. On one hand, consistent with prior research findings (Ayala and Manzano, 2018), emotional intelligence positively predicts psychological resilience in EFL learners. Individuals with high emotional intelligence can more effectively identify and manage their own and others’ emotions, thereby laying the foundation for the development of psychological resilience. Through improving emotional states, e.g., reducing anxiety, and promoting objective assessments of task difficulty, e.g., cognitive restructuring, emotional intelligence enhances behavioral willingness. Simultaneously, the psychological resources accumulated by high emotional intelligence—such as an optimistic mindset—translate into more adaptive coping strategies, e.g., time management techniques (Solomon and Rothblum, 1984; Wang and Li, 2021). This efficacy in emotional regulation directly strengthens psychological resilience, enabling individuals to maintain stable mental states when encountering academic pressure, thereby forming a positive feedback loop of “emotional resilience-behavioral control.” On the other hand, psychological resilience negatively predicts academic procrastination among EFL learners, supporting prior research findings (Ayala and Manzano, 2018; Miraj et al., 2021) that psychological resilience inhibits academic procrastination. According to the Self-regulatory Executive Function Theory, psychological resilience helps individuals avoid using procrastination to escape emotional distress (Matthews and Wells, 2000). When facing academic pressure, resilience operates through dual mechanisms—the compensatory model and the protective factor model, that is, the psychological resilience not only independently counteracts the negative effects of stress, but enhances perceived control over tasks, thereby reducing the likelihood of procrastination, which indicates that psychological resilience serves as a key protective factor against the high-pressure and procrastinated vicious cycle. Consequently, enhancing psychological resilience is an effective approach to mitigating academic procrastination and should be integrated into the core competency framework for student development.

### 4.3 Relationship between emotional intelligence and academic procrastination serially mediated by self-efficacy and psychological resilience

Further research reveals that self-efficacy serves as a key promotive factor of psychological resilience. These two constructs synergistically constitute the intrinsic mechanism through which emotional intelligence influences academic procrastination. Specifically, emotional intelligence indirectly enhances psychological resilience by strengthening an individual’s self-efficacy beliefs, ultimately leading to a significant reduction in academic procrastination due to the buffering effect of psychological resilience. This suggests that understanding students’ academic procrastination requires a comprehensive grasp of their intrinsic traits and abilities at cognitive, emotional, and behavioral levels. The present study focuses on the interactive mechanisms among learners’ multidimensional psychological traits. Empirical results validate a dynamic pathway through which these traits collaboratively influence academic procrastination. Emotional intelligence—emphasizing the ability to identify, understand, manage, and utilize emotions—enhances attentional and cognitive functions by promoting de-automatization, reducing rumination, countering negative thinking, and mitigating avoidance behaviors. In contrast, self-efficacy centers on an individual’s belief in their capability to successfully complete specific tasks. Emotional intelligence thus indirectly affects academic procrastination by shaping self-efficacy. When students possess high emotional intelligence, they can better regulate their emotions when confronting academic tasks, preventing self-doubt, anxiety, or fear from dominating their responses. Simultaneously, EFL learners’ psychological resilience becomes strengthened, enabling them to demonstrate greater perseverance when confronting academic difficulties and setbacks rather than being easily defeated by adversity, they maintain a proactive attitude and actively seek solutions instead of resorting to procrastination. Furthermore, this study reveals that self-efficacy exhibits significantly stronger explanatory power compared to the indirect effect of psychological resilience. Specifically, self-efficacy not only directly negatively predicts academic procrastination but also retains critical regulatory functions within the serial pathway of ‘emotional intelligence → psychological resilience’. When cumulative academic tasks intensify learning pressure, students must reinforce self-efficacy beliefs to enhance their resilience. This confidence in overcoming academic setbacks and adapting to learning environments translates into higher levels of academic resilience, ultimately helping them break through learning impasses. These findings confirm that self-efficacy acts as a protective barrier for psychological resilience, further solidifying its theoretical positioning as a core driver of resilience. Moreover, enhanced psychological resilience empowers learners to better resist external environmental distractions. Although EFL learners generally exhibit high emotional intelligence, certain individuals may experience reduced levels due to external stressors or deficient self-regulation. Such learners must prioritize improving their emotional intelligence. This serial effect illuminates the interrelationships among emotional management, self-belief, and psychological resilience in the learning process, offering novel perspectives and methods for understanding and resolving academic procrastination. These insights hold significant implications for educational practice.

## 5 Conclusion

The present study, through empirical analysis, pined the combined influence mechanism of emotional intelligence, self-efficacy, and psychological resilience on academic procrastination among EFL learners in China. Data analysis indicates that emotional intelligence, self-efficacy, and psychological resilience all significantly and negatively predict EFL learners’ level of academic procrastination. Further mediation effect tests show that self-efficacy and psychological resilience each play a partial mediating role between EFL learners’ emotional intelligence and academic procrastination, and together they form a serial mediation pathway. From the perspective of individual intrinsic psychological resources, these results clarify the causal mechanisms of academic procrastination. They suggest that systematically cultivating students’ emotional intelligence, strengthening self-efficacy, and enhancing psychological resilience can effectively reduce academic procrastination behaviors. This provides new directions for educational interventions targeting academic procrastination in higher education. However, this study relied solely on self-reported cross-sectional data from students, which fails to capture the dynamic relationships among these variables. Additionally, it primarily focused on internal psychological capital while insufficiently exploring the impact of external environmental factors. All in all, this study is among the first steps to uncovering the reciprocal relationships between these variables. As it seems, this domain is still in its infancy and asks for more empirical studies to brighten the road, and future research should prioritize longitudinal studies to further investigate the influence pathways of family, school, and other social environmental factors on emotional intelligence, self-efficacy, and academic procrastination, which will provide more targeted intervention strategies for educational practice.

## Data Availability

The raw data supporting the conclusions of this article will be made available by the authors, without undue reservation.

## Footnotes

1
https://www.wjx.cn
